# Association between Long-Term Exposure to Traffic-Related Air Pollution and Subclinical Atherosclerosis: The REGICOR Study

**DOI:** 10.1289/ehp.1205146

**Published:** 2012-12-12

**Authors:** Marcela Rivera, Xavier Basagaña, Inmaculada Aguilera, Maria Foraster, David Agis, Eric de Groot, Laura Perez, Michelle A. Mendez, Laura Bouso, Jaume Targa, Rafael Ramos, Joan Sala, Jaume Marrugat, Roberto Elosua, Nino Künzli

**Affiliations:** 1 Centre for Research in Environmental Epidemiology (CREAL), Barcelona, Spain; 2 Hospital del Mar Research Institute (IMIM), Barcelona, Spain; 3 Universitat Pompeu Fabra, Barcelona, Spain; 4 University of Montreal Hospital Research Centre (CRCHUM), Montreal, Quebec, Canada; 5 CIBER Epidemiología y Salud Pública (CIBERESP), Spain; 6 Department of Vascular Medicine, Academic Medical Centre, Amsterdam, the Netherlands; 7 Swiss Tropical and Public Health Institute, Basel, Switzerland; 8 University of Basel, Basel, Switzerland; 9 University of North Carolina at Chapel Hill, Chapel Hill, North Carolina, USA; 10 4sfera Innova, Girona, Spain; 11 Research Unit, Family Medicine, Jordi Gol Institute for Primary Care Research (IDIAP Jordi Gol), Catalan Institute of Health, Catalunya, Spain; 12 Department of Medical Sciences, School of Medicine, University of Girona, Girona, Spain; 13 Servicio de Cardiología, Hospital Universitari Josep Trueta, Institut Català de la Salut, Girona, Spain; 14 Grupo de Epidemiología y Genética Cardiovascular, Hospital del Mar Research Institute (IMIM), Barcelona, Spain

**Keywords:** ankle–brachial index, average daily traffic, cardiovascular disease, exposure assessment, exposure to tailpipe emissions, intima media thickness, land use regression model, Mediterranean diet, nitrogen dioxide

## Abstract

Background: Epidemiological evidence of the effects of long-term exposure to air pollution on the chronic processes of atherogenesis is limited.

Objective: We investigated the association of long-term exposure to traffic-related air pollution with subclinical atherosclerosis, measured by carotid intima media thickness (IMT) and ankle–brachial index (ABI).

Methods: We performed a cross-sectional analysis using data collected during the reexamination (2007–2010) of 2,780 participants in the REGICOR (Registre Gironí del Cor: the Gerona Heart Register) study, a population-based prospective cohort in Girona, Spain. Long-term exposure across residences was calculated as the last 10 years’ time-weighted average of residential nitrogen dioxide (NO_2_) estimates (based on a local-scale land-use regression model), traffic intensity in the nearest street, and traffic intensity in a 100 m buffer. Associations with IMT and ABI were estimated using linear regression and multinomial logistic regression, respectively, controlling for sex, age, smoking status, education, marital status, and several other potential confounders or intermediates.

Results: Exposure contrasts between the 5th and 95th percentiles for NO_2_ (25 µg/m^3^), traffic intensity in the nearest street (15,000 vehicles/day), and traffic load within 100 m (7,200,000 vehicle-m/day) were associated with differences of 0.56% (95% CI: –1.5, 2.6%), 2.32% (95% CI: 0.48, 4.17%), and 1.91% (95% CI: –0.24, 4.06) percent difference in IMT, respectively. Exposures were positively associated with an ABI of > 1.3, but not an ABI of < 0.9. Stronger associations were observed among those with a high level of education and in men ≥ 60 years of age.

Conclusions: Long-term traffic-related exposures were associated with subclinical markers of atherosclerosis. Prospective studies are needed to confirm associations and further examine differences among population subgroups.

Air pollution from traffic and other sources is an established cause of premature mortality (Brook et al. 2010). A relevant part of this environmental burden of disease relates to cardiovascular diseases (CVDs), which were responsible for 10% of total disability-adjusted life years in 2004 and the leading causes of death worldwide in 2008 (World Health Organization 2008). A common feature of this group of diseases is atherosclerosis, a chronic and degenerative process that mainly occurs in large and medium-sized arteries and is characterized by asymmetric focal thickenings of the innermost layer of the artery, the intima. The development of atherosclerosis is the result of the total cumulative exposure to atherogenic risk factors such as hypertension, high cholesterol, diabetes, obesity, smoking, physical inactivity, and other lifestyle factors and their interactions with genetic susceptibility (Lusis 2000). Acute events such as myocardial infarction or stroke can be triggered by short-term exposure to air pollution (Brook et al. 2010). However, whether and how ambient air pollution contributes to atherogenesis is subject to debate. Although experimental studies on animals provide strong evidence for a causal atherogenic role of air pollution, particularly in obese mice (Sun et al. 2005), evidence from epidemiological studies is limited.

The long subclinical phase of atherosclerosis makes it possible to investigate the determinants of the vascular pathology long before its clinical manifestation. The association between air pollution and intima media thickness (IMT), an established marker of subclinical atherosclerosis, was reported for the first time in volunteers participating in two clinical trials in California (Künzli et al. 2005). Two population-based cross-sectional analyses, namely the Heinz Nixdorf Recall study in Germany (Bauer et al. 2010) and the Multi-Ethnic Study of Atherosclerosis (MESA) cohort in the United States (Diez Roux et al. 2008), also reported associations between air pollution and IMT, although a study of young adults in the Netherlands found no association (Lenters et al. 2010). So far, only one longitudinal study has been published, and it was based on heterogeneous samples of volunteers participating in five clinical trials (Künzli et al. 2010), including the two trials of the first cross-sectional study published on this topic (Künzli et al. 2005). Findings from the study suggested a possible role of ambient air pollutants, indicated by particulate matter (PM) ≤ 2.5 µm in diameter (PM_2.5_) and living close to busy highways, in the progression of IMT (Künzli et al. 2010).

Most previous studies have reported that associations between air pollution and IMT differ among population subgroups (Künzli et al. 2011). However, subgroup patterns have not been consistent; thus a clear understanding of susceptibility factors is still lacking. Human studies have not investigated whether diet modifies the effects of air pollution—a plausible hypothesis given evidence from animal studies (Sun et al. 2005) and the effects of diet on oxidative stress and systemic inflammation that are likely to contribute to the systemic effects of ambient air pollution (Brook et al. 2010).

The Mediterranean region of Girona, and Spain in general, has one of the lowest cardiovascular mortality rates in Europe, despite very high prevalence of conventional cardiovascular risk factors (Masiá et al. 1998), a paradox that may be explained in part by protective effects of the Mediterranean diet (Martínez-González et al. 2011). We aimed to investigate the association between long-term exposure to traffic-related air pollution and subclinical atherosclerosis in Spain, and modification of this association by diet and other factors identified in previous studies (Künzli et al. 2011). We investigated this in the follow-up of participants of three population-based cohorts of the REGICOR (Registre Gironí del Cor: the Gerona Heart Register) study (Grau et al. 2007). Subclinical atherosclerosis was measured by carotid IMT, a validated marker of atherosclerosis (Coll and Feinstein 2008). In addition, we measured the ankle–brachial index (ABI), a marker of the presence and severity of peripheral artery disease. Both IMT and ABI are associated with cardiovascular events and mortality (Ankle Brachial Index Collaboration 2008; Lorenz et al. 2007). In the absence of PM measurements in this region, we used estimates of 10-year average home outdoor nitrogen dioxide (NO_2_) concentrations and residential traffic intensity as markers of exposure to local traffic-related air pollutants.

## Methods

REGICOR-Air is a cross-sectional study nested in the REGICOR cohort study (Grau et al. 2007) evaluating the association between air pollution and cardiovascular diseases. We used data from the follow-up of three population-based cohorts originally enrolled in 1995, 2000, and 2005 (with response rates of 72.4, 70.0, and 73.8%, respectively) from 12 towns that represent the geographic diversity of the Girona Province and have large contrasts in ambient air pollution levels [see map in Supplemental Material, Figure S1 (http://dx.doi.org/10.1289/ehp.1205146)]. During 2007–2010, the participants residing in these towns, who were alive and not institutionalized, were invited to participate in REGICOR-Air, and the response rate was approximately 82%. In addition to extensive health status reassessment, IMT and ABI were measured for the first time in this population. Address histories for the past 10 years were collected by questionnaire, and each address was geocoded at the front-door level. This study was approved by the Hospital del Mar Research Institute ethics committee, and participants gave written informed consent.

IMT was measured by three trained, certified sonographers using ultrasound examinations of the left and right carotid arteries. Standardized scan and image analyses protocols were used. The scan protocol entailed the right and left carotid arteries. In each artery, the following predefined longitudinal segments were imaged: *a*) the common carotid 1 cm proximal to the dilation, *b*) the carotid bulb, and *c*) the internal carotid 1 cm distal to the flow divider. For imaging, an Acuson Aspen ultrasound instrument (Acuson-Siemens, Erlangen, Germany) equipped with an L7 5-12MHz transducer and dedicated REGICOR application scan protocol (AMC IMAGELAB, University of Amsterdam, Amsterdam, and Technical University Eindhoven, Eindhoven, the Netherlands) were used. A still image of each arterial segment was saved as a DICOM (digital information and communication in medicine) file. These source files were locally stored and securely transferred to IMAGELAB, where trained and certified sonographers analyzed the images using validated “eTrack REGICOR” software (Department of Physiology and Vascular Medicine Academic Medical Centre, Amsterdam, the Netherlands) (de Groot et al. 2008). IMT was defined as the average distance between the lumen-intima and media-adventitia interfaces in a given 1 cm segment of the artery far wall. The main outcomes were *a*) the mean of the IMTs for the left and right common carotid arteries (IMTcca), and *b*) the mean of the IMTs for the left and right common carotid arteries, internal carotid arteries, and carotid bulbs (IMT6seg). These two outcomes were assessed separately because of differences in the segments’ cellular constituents—with more foam cells in the common carotid artery and more cholesterol-rich plaques in the carotid bulb and the internal carotid artery (Gállego Pérez-Larraya et al. 2012)—and in risk factors for IMT (Polak et al. 2010), which suggest that etiology may differ according to location. Between-sonographer and between-visit variability were evaluated based on repeated IMT measurements conducted at two visits, 2 weeks apart, by up to three sonographers, in 42 participants.

Systolic blood pressure was measured in a supine position after a 5-min rest in the brachial artery of both arms and the posterior tibial and dorsalis pedis arteries of both legs, using a continuous Doppler device. Right and left ABI were calculated as the ratio of the highest leg pressure to the highest brachial pressure in the corresponding arm, and the lowest of the two ABI ratios were categorized as low (< 0.9), normal (0.9–1.3), or high (> 1.3) for analysis (Ankle Brachial Index Collaboration 2008; McDermott et al. 2005).

Adherence to the Mediterranean diet was measured by a 10-point index based on sex-specific intake tertiles of seven beneficial (cereals, fruits, vegetables, legumes, seafood, nuts, moderate red wine) and two detrimental (meats, dairy products) food groups, and categorized in quartiles. Modifications such as excluding low-fat dairy products or white meats from the detrimental foods, or incorporating additional unhealthy food groups (e.g., soft drinks, salty snacks, pastries) as detrimental components, had no meaningful impact on findings in sensitivity analyses (data not shown). A detailed description of the methodology used to measure adherence to the Mediterranean diet in the REGICOR study is given by Schröder et al. (2004). The plausibility of reported dietary intakes was assessed based on disparities between reported energy intakes and estimated energy requirements (Mendez et al. 2011).

Participants with a clinical history of CVD (myocardial infarction, stroke, angina, catheterization, angioplasty, bypass surgery, or amputation due to circulatory problems) were excluded (*n* = 227) because medication use or altered health behaviors among these participants may have influenced IMT/ABI measures obtained for this study.

*Exposure assessment.* We estimated the 10-year time-weighted average of the home outdoor concentrations of NO_2_ for each participant using land use regression (LUR) models. In the absence of air quality data in most of these towns, we conducted an extensive monitoring campaign using NO_2_ passive samplers to validate model estimates. We measured NO_2_ in the balcony of 562 participants’ homes for 1 month in the spring and again in the fall between June 2007 and July 2009. Homes were selected to cover a broad range of traffic-related pollution and urban settings (e.g., low- and high-building–density areas), to be representative of the residential locations of the cohort participants and to be well distributed across the towns. We adjusted for temporal variability using monthly mean NO_2_ concentrations collected at a fixed location in each town for at least 1 year. NO_2_ annual means were derived by multiplying the monthly means at each location by the ratio of the annual mean to the same month mean NO_2_ at the town’s fixed location.

To predict NO_2_ at each participant’s residence we used LUR models based on NO_2_ annual means and data on traffic intensity, bus routes and stops, distance to traffic, land cover, building density, and other geographic information system (GIS)-derived variables. Given the geographic diversity of the study area and differences in the availability of GIS data among towns, we divided the study area into seven subareas, i.e., groups of adjacent towns. LUR models were derived for each group by supervised forward linear regression according to the methodology described by Rivera et al. (2012). The models explained between 33% and 63% of measured NO_2_ (cross-validation *R*^2^ 0.32 and 0.61, respectively) [see Supplemental Material, Table S1 and Figure S2 (http://dx.doi.org/10.1289/ehp.1205146)].

We estimated the outdoor annual mean NO_2_ at each residential location by applying the LUR models to the address geocode. NO_2_ concentrations were back-extrapolated to the time period of residence in each address using daily means at a fixed urban background monitoring location. Finally, for each participant we calculated the time-weighted average of NO_2_ estimates across all residences in the 10 years preceding the IMT measurement (10-year NO_2_ exposure). Time periods when participants lived at addresses that were geocoded with low precision or were outside of the study area were excluded when deriving the 10-year NO_2_ exposure. Participants who lived in the study towns for < 6 years or had high-precision geocodes (i.e., at the front-door level) for < 6 years (*n* = 365) were excluded from the main analyses.

We also used traffic proximity markers as surrogates of air pollution exposure in independent analyses. Traffic intensity, available from local registries and the Urban Mobility Plan for Girona (2007), was assigned to the central road network used within ESCAPE (European Study of Cohorts for Air Pollution Effects) (Hoek et al. 2012). Traffic counts were conducted at approximately 670 streets with missing traffic information to complete the traffic inventory. The traffic intensity assessment is described in detail elsewhere (Rivera et al. 2012). For each address, we calculated the traffic intensity at the nearest street and the traffic load (sum of traffic intensity multiplied by length of road segment in all segments) in a 100 m buffer and derived 10-year average values for each participant.

All GIS calculations were performed using ArcGIS 9.2 (ESRI, Redlands, CA, USA).

*Statistical analysis.* Crude and adjusted associations of IMT (which were natural log-transformed to reduce heteroscedasticity) with individually assigned air pollution exposures were estimated using linear regression models. Linear models were compared with additive models with smoothing splines using the generalized additive model (GAM) function in the MGCV R package (http://www.R-project.org) and linearity was confirmed. We analyzed ABI using multinomial logistic regression (Hosmer and Lemeshow 2004). We initially adjusted our models by age and sex. Age was a main determinant of both outcome and exposure. Thus, in a second step, we analyzed the association of the potential confounders with both outcome and exposure variables, adjusting by age and sex. Variables associated with both, namely, smoking status, education level (as a proxy of socioeconomic status), and marital status were included in a minimal adjustment set. The association between exposure and IMT was strongly confounded by age. To determine whether age was an effect modifier, the interaction term age × exposure was introduced in the model, and it was not statistically significant (*p* > 0.05). We also evaluated whether the effect of age differed for men and women. This was the case, so we added the sex × age interaction to our minimal adjustment set (model 1). We further adjusted by other potential confounders {physical activity [weekly energy expenditure in leisure time based on the Minnesota questionnaire (Elosua et al. 2000) expressed in tertiles of metabolic equivalents], diet [adherence to Mediterranean diet and plausibility of reported diet], SES at the census-tract level [percentage of residents with < secondary school education in the tract where participants resided the longest, based on the 2001 census], and occupational status}, and results (not shown) did not differ from those of model 1. Finally, in addition to the variables listed above, we also included potential intermediates [body mass index (BMI), high-density lipoprotein level (HDL), waist circumference, systolic and diastolic blood pressure, and medication treatment (lipid-lowering, antihypertensives, antiplatelets, or anticoagulants, based on self-report and medical records)] to explore the effects of air pollution that do not follow these pathways (model 2). Adjustment variables were entered in the models either as continuous variables or dummy variables for all categories according to Table 1. [Also see Supplemental Material, Table S2 (http://dx.doi.org/10.1289/ehp.1205146).]

**Table 1:** Descriptive statistics [*n* (%), unless otherwise indicated] of the study population characteristics included in main analyses (*n* = 2,780).

Characteristic	Value
IMTcca [mm (median ± IQR)]	0.68 ± 0.19
IMT6seg [mm (median ± IQR)]	0.67 ± 0.18
ABI (lower of left and right)
ABI < 0.9	56 (2.0)
ABI > 1.3	116 (4.2)
Age [years (median ± IQR)]	58 ± 18
Sex (women)	1,491 (53.6)
Education level
Primary school or illiterate	1,476 (53.1)
Secondary school	758 (27.3)
Technician or higher education degree	526 (18.9)
Occupational status
Employed	1,447 (53.1)
Inactive or housekeeper	358 (12.9)
Retired	852 (30.7)
Unemployed	68 (2.5)
Smoking status
Never smoker	1,202 (54.5)
Former smoker	628 (28,5)
Current smoker	377 (17.1)
Marital status
Single	165 (5.9)
Married/living together	2,178 (78.4)
Divorced	171 (6.2)
Widow	247 (8.9)
Other	9 (0.3)
BMI [kg/m2 (median ± IQR)]	26.6 ± 5.5
Waist circumference [cm (median ± IQR)]	93 ± 17
HDL [mg/dL (median ± IQR)]	52.9 ± 15.6
Any cardiovascular or antihypertensive medication treatment	1,137 (40.9)
Mediterranean diet index (median ± IQR)	25 ± 4
Energy expenditure in leasure time [MET-min/week (median ± IQR)]	1,515 ± 1,937
People with low education in the census tract [% (median ± IQR)]	11 ± 12.2
Living at the same address for 10 years before IMT measurement	2,252 (81)
Abbreviations: IQR, interquartile range; MET, metabolic equivalent.	

We did not adjust the exposure–outcome associations for area of residence in the main analyses because the study area was relatively small (65 × 70 km), data collection in every town was done by the same team using the exact same procedures and because doing so would have partially removed the exposure contrast corresponding to between-town variability. However, we explored the sensitivity of the results to the inclusion of area of residence (corresponding to the address of longest residence) as a random effect variable. The trend of the associations remained the same, but the estimates were closer to the null and less precise (data not shown). This is consistent with a decrease in the exposure contrast, which makes the detection of an effect more difficult. Residual confounding by area is less likely because the estimates were adjusted by a large array of confounders, including area-level confounders such as education at the census-tract level.

Because living near busy roads is also associated with traffic-related noise, we adjusted for road traffic noise in a subsample of participants with available noise data (*n* = 1,084) (Foraster et al. 2011). However, results were not sensitive to adjustment for noise (data not shown).

Results are expressed as the cross-sectional percent difference in IMT, and the relative risk ratio (RRR) of high and low ABI, associated with a 10-year exposure contrast corresponding to the difference between the 5th and 95th percentiles in the study population. The RRR of high ABI is the prevalence of high ABI relative to normal ABI in exposed participants divided by the prevalence of high ABI relative to normal ABI in nonexposed participants.

We tested effect modification by diet, and factors identified as potential effect modifiers in previous studies, namely, age, sex, education level, smoking, diabetes, menopause, and medication treatment, using stratified analysis. We report the effect estimates of percent differences in IMT by strata of the potential effect modifiers.

To assess the sensitivity of the results to the NO_2_ LUR models, we used the annual mean NO_2_ at the closest monitor both within 100 m and within 200 m of the residence, instead of the 10-year modeled NO_2,_ in two separate sensitivity analyses. Median distance to the closest NO_2_ monitoring site was 90 m. The sample size was restricted in these analyses because only those whose address of longest residence was within 100 m and within 200 m of a monitor were kept (e.g., 2,265 and 1,778 persons lived within 200 m of a monitor with data available on IMTcca and IMT6seg, respectively).

Analyses were performed using Stata version 10.1 (StataCorp, College Station, TX, USA) and R version 2.12 (http://www.R-project.org). The alpha level for statistical significance was set at 0.05.

## Results

Information on IMTcca, IMT6seg, and ABI was available for 2,780, 2,188, and 2,738 participants, respectively. Fewer participants had IMT6seg measurements because of difficulties in analyzing the images to obtain measurements from the internal carotid artery and carotid bulb (Gállego Pérez-Larraya et al. 2012). The characteristics of the study populations are summarized in Table 1. Participants were 32–86 years of age. Percentages of participants with low, medium, and high education levels are consistent with Spain as a whole (Instituto Nacional de Estadística 2012). Participants included in the analyses did not differ from those excluded (participants who lived in the study towns for < 6 years or with clinical history of CVD) in terms of exposure levels (data not shown). The median IMTcca was 0.68 mm (range, 0.40–2.05 mm). The repeatability study showed intraclass correlation coefficients for sonographers and visits of 0.83 for the IMTcca and of 0.77 for the IMT6seg. ABI was on average 1.10 (range, 0.5–1.75) with 2.0% of the study population classified as having low ABI (< 0.9), and 4.2% with high ABI (> 1.3) (Table 1). Participants with low ABI were on average 9 years older than participants with normal ABI, whereas those with high ABI did not differ in age from participants with normal ABI (data not shown). Inter- and intraoperator variability of ABI measurements were low, with intraclass correlation coefficients of 0.92 and 0.94, respectively.

The 10-year average home outdoor NO_2_ concentrations varied from 5 to 48 µg/m^3^ [see Supplemental Material, Table S3 (http://dx.doi.org/10.1289/ehp.1205146)], and its correlation with NO_2_ at the address of longest residence was > 0.99 over all participants (see Supplemental Material, Table S4) and 0.96 among those who moved at least once during the 10 years (data not shown). Ranges of 10-year time-weighted average values for traffic in the nearest street and traffic within 100 m indicated substantial variability among participants overall (see Supplemental Material, Table S3) and according to town of residence (see Supplemental Material, Table S5). Traffic intensity showed higher spatial variability than NO_2_ [(75th percentile – 25th percentile)/50th percentile was 2.51 for 10-year traffic in the nearest street, 1.56 for 10-year traffic load in 100 m, and 0.73 for 10-year NO_2_]. NO_2_ and traffic exposure variables were moderately correlated (Pearson correlation coefficients, *r* = 0.52–0.72), and the two traffic variables had a correlation of 0.58 (see Supplemental Material, Table S4).

In unadjusted models, NO_2_, traffic in the nearest street, and traffic in the 100 m buffer were strongly associated with the intima media thickness (both IMTcca and IMT6seg) (Table 2). Associations decreased after adjusting for age, and in general, further adjustment (model 1 and model 2) provided similar estimates. Associations between a 25 μg/m^3^ increase in NO_2_ (corresponding to the difference between the 5th and 95th percentiles of exposure) and both IMTcca and IMT6seg were positive, but small and nonsignificant after adjustment. Fully adjusted models—including potential intermediates (model 2)—indicated that a corresponding exposure contrast for traffic load within 100 m (7,200,000 vehicle-m/day) was associated with a 1.91% difference in IMTcca (95% CI: –0.24, 4.06) and a 2.06% difference in IMT6seg (95% CI: –0.09, 4.21). An increase of 15,000 vehicles/day on the nearest street was associated with a 2.32% difference in IMTcca (95% CI: 0.48, 4.17) and a 1.80% difference in IMT6seg (95% CI: 0.01, 3.59).

**Table 2:** Estimated percent difference in IMT associated with a 10-year average exposure contrast between the 5th and 95th percentiles.

Exposure (exposure contrast)	IMTcca		IMT6seg	
	n	% change (95% CI)	n	% change (95% CI)
NO2 (25 µg/m3)
Crude	2,780	3.67 (1.37, 5.98)	2,188	4.98 (2.65, 7.31)
Adjusted for sex	2,780	3.67 (1.38, 5.96)	2,188	4.88 (2.58, 7.18)
Adjusted for age × sex	2,780	0.04 (–1.83, 1.92)	2,188	0.84 (–1.02, 2.71)
Model 1a	2,738	0.35 (–1.63, 2.32)	2,155	0.71 (–1.25, 2.67)
Model 2b (possible intermediates)	2,632	0.56 (–1.47, 2.59)	2,074	0.52 (–1.52, 2.57)
Traffic load in a 100 m bufferc (7,200,000 veh-m/day)
Crude	2,780	5.25 (2.76, 7.74)	2,188	6.38 (3.89, 8.88)
Adjusted for sex	2,780	5.21 (2.73, 7.68)	2,188	6.31 (3.85, 8.78)
Adjusted for age × sex	2,780	1.39 (–0.64, 3.42)	2,188	1.99 (–0.02, 4)
Model 1	2,738	1.78 (–0.33, 3.89)	2,155	2.08 (0, 4.17)
Model 2 (possible intermediates)	2,609	1.91 (–0.24, 4.06)	2,053	2.06 (–0.09, 4.21)
Traffic intensity in nearest street (15,000 veh/day)
Crude	2,780	4.18 (2.01, 6.35)	2,188	4.55 (2.43, 6.68)
Adjusted for sex	2,780	4.13 (1.98, 6.29)	2,188	4.42 (2.32, 6.51)
Adjusted for age × sex	2,780	1.74 (–0.02, 3.5)	2,188	1.75 (0.05, 3.44)
Model 1	2,738	1.96 (0.14, 3.77)	2,155	1.7 (–0.04, 3.44)
Model 2 (possible intermediates)	2,632	2.32 (0.48, 4.17)	2,074	1.8 (0.01, 3.59)
veh, vehicle. aEstimates adjusted by model 1: sex, age, sex–age interaction, smoking status, education, and marital status. bEstimates adjusted by model 2: model 1 plus BMI, HDL, waist circumference, systolic and diastolic blood pressure, weekly energy expenditure in physical activity during leisure time (tertiles), adherence to Mediterranean diet, plausibility of reported diet, medication treatment, and percentage of persons with low education at the census-tract level. cModels for traffic load were additionally adjusted for occupational status.							

Ten-year NO_2_ and both residential traffic indicators were associated with higher prevalence of high ABI (Table 3). Adjusted RRRs for high versus normal ABI were 1.98 (95% CI: 1.09, 3.60) for a 25 µg/m^3^ increase in NO_2_; 1.89 (95% CI: 1.07, 3.34) for a 7,200,000 vehicle-m/day increase in traffic load within 100 m; and 1.70 (95% CI: 1.13, 2.57) for an increase of 15,000 vehicles/day on the nearest street. The RRR of low ABI compared to normal ABI were consistent with the null hypothesis for all exposures.

**Table 3:** RRRs (95% CIs) for low or high ABI (< 0.9 or > 1.3 versus ABI = 0.9 to 1.3, respectively) associated with 10-year average exposure contrasts between the 5th and 95th percentiles.

Exposure (exposure contrast)	n	ABI < 0.9			ABI > 1.3	
NO2 (25 µg/m3)
Model 1a	2,698	0.65	(0.28, 1.52)	1.91	(1.09, 3.33)
Model 2b (possible intermediates)	2,600	0.72	(0.29, 1.75)	1.98	(1.09, 3.6)
Traffic load in a 100 m bufferc (7,200,000 veh-m/day)
Model 1	2,698	0.92	(0.37, 2.3)	1.89	(1.1, 3.26)
Model 2 (possible intermediates)	2,600	1.02	(0.4, 2.61)	1.89	(1.07, 3.34)
Traffic intensity in nearest street (15,000 veh/day)
Model 1	2,698	0.47	(0.16, 1.41)	1.6	(1.08, 2.38)
Model 2 (possible intermediates)	2,600	0.48	(0.16, 1.46)	1.7	(1.13, 2.57)
veh, vehicle. aEstimates adjusted by model 1: sex, age, sex–age interaction, smoking status, education, and marital status. bEstimates adjusted by model 2: sex, age, sex–age interaction, smoking status, education, marital status, BMI, HDL, waist circumference, systolic and diastolic blood pressure, weekly energy expenditure in physical activity during leisure time (tertiles), adherence to Mediterranean diet, plausibility of reported diet, medication treatment, and percentage of persons with low education at the census-tract level. cModels for traffic load were additionally adjusted for occupational status.						

**Figure 1:**
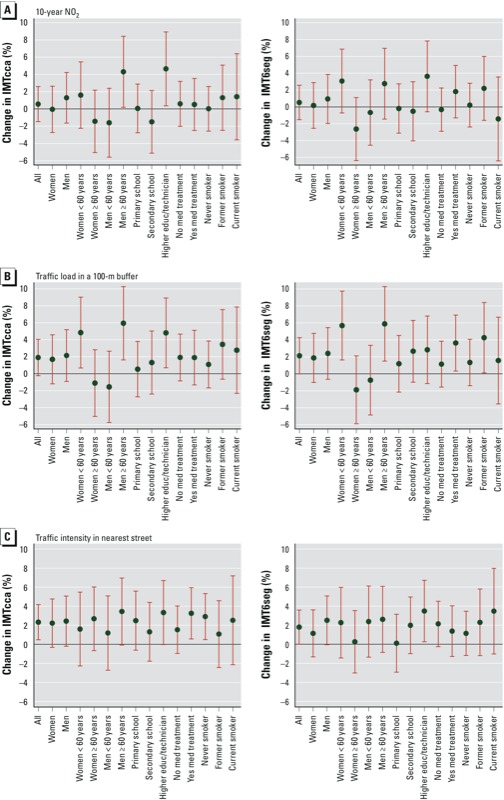
Subgroup analyses: estimates of percent differences (95% CIs) in IMTcca (left) and IMTseg (right) associated with exposure contrasts between the 5th and 95th percentiles for 10-year weighted average values of NO_2_ (25 µg/m^3^; *A*), traffic load within 100 m (7,200,000 vehicle-m/day; *B*), and traffic intensity on the nearest street (15,000 vehicles/day; *C*) according to sex, sex × age ≥ 60 years, education, medication treatment, and smoking status. Estimates adjusted by sex, age, sex × age interaction, smoking status, education, marital status, BMI, HDL, waist circumference, systolic and diastolic blood pressure, weekly energy expenditure in physical activity during leisure time (tertiles), adherence to Mediterranean diet, plausibility of reported diet, medication (med) treatment, and percentage of people with low education (educ) at the census-tract level. Models for traffic load were additionally adjusted for occupational status.

*Effect modification.* The association of all exposure markers with IMT differed across education level (Figure 1). In persons with a higher level of education, the association of 10-year exposure to air pollution with IMT was stronger (model 2 with NO_2_ and IMTcca). Increases of 25 μg/m^3^ in NO_2_, 7,200,000 vehicle-m/day in traffic load in 100 m, and 15,000 vehicles/day on the nearest street were associated with 4.6% (95% CI: 0.4, 8.9)_,_ 4.8% (95% CI: 0.7, 8.9), and 3.3% (95% CI: –0.02, 6.7) differences in IMTcca, respectively, among persons with a high education level; –1.5% (95% CI: –5.1, 2.1), 1.3% (95% CI: –2.4, 5.0), 1.3% (95% CI: –1.8, 4.4) among persons with a secondary school–level education; and 0.6% (95% CI: –2.8, 2.9), 0.5% (95% CI: –2.7, 3.8), 2.4% (95% CI: –0.6, 5.6) among persons with a primary school–level education. Similar patterns were observed for the associations of exposure to air pollutants with IMT6seg. Effects estimates were also stronger in men ≥ 60 years of age compared to younger men, showing significant associations of NO_2_ and traffic load in 100 m with IMT (percent difference in IMTcca for NO_2_, traffic load, and traffic intensity were 4.3% (95% CI: 0.2, 8.4), 5.9% (95% CI: 1.6, 10.3), and 3.4% (95% CI: –0.07, 7.0), respectively, among men ≥ 60 years of age, and –1.5% (95% CI: –5.7, 2.7), –1.5% (95% CI: –5.7, 2.6), and 1.2% (95% CI: –2.7, 5.0), respectively, among men < 60 years of age. No evidence of effect modification by smoking, medication treatment (Figure 1), menopause, diabetes (results not shown), or Mediterranean diet [see Supplemental Material, Figure S3 (http://dx.doi.org/10.1289/ehp.1205146)] was observed.

## Discussion

Our multivariate models revealed positive associations between three markers of long-term exposure to traffic-related air pollution, namely traffic load within 100 m of the residence, traffic intensity in the nearest street, and modeled concentration of home outdoor NO_2_ and carotid subclinical atherosclerosis in a random healthy (no history or current signs of CVD) population sample of the Spanish Mediterranean region. However, associations with NO_2_ were weak and reached statistical significance only in the subgroups of persons with a high education level (IMTcca and IMT6seg) and men ≥ 60 years of age (IMTcca). Both indicators of residential traffic were associated with approximately 2% thicker carotid intima-media. High exposures to NO_2_ and traffic were also associated with higher prevalence of high ABI [RRR for high vs. normal ABI between 1.70 (95% CI: 1.13, 2.57) for traffic intensity in nearest street and 1.98 (95% CI: 1.09, 3.60) for NO_2_]. To put these results in context with common risk factors for atherosclerosis, in the same study population included in main analyses, a 10-year difference in age, adjusted by confounders in model 2, was associated with an 8% (95% CI: 7.3, 9.0) difference in IMTcca and an RRR of 10.3 (95% CI: 10.0, 10.5) for high versus normal ABI. Interestingly, age strongly confounded the association of pollution with IMT. Removing age adjustment from the main multivariate models resulted in substantially larger estimates, very similar to the crude associations. Atherosclerosis is a lifetime process, thus age is a strong correlate of the cumulated lifetime exposure to air pollution as well as the main predictor of atherosclerosis. Therefore, adjustment for age may in part reflect overadjustment for exposure.

Our results suggest that long-term exposure to traffic-emitted pollution may contribute to carotid artery atherosclerosis. Given widespread exposure to high traffic intensity, the potential association of long-term pollution with atherosclerosis has important public health implications. Ninety-five percent of our study population was exposed to traffic intensity levels in the nearest street of < 15,000 vehicles/day, which is very low compared with other European cities. For example, in larger cities such as Barcelona, many people live within very short distances of streets with traffic intensities of 50,000–100,000 vehicles/day (Amato et al. 2009). Likewise, levels of NO_2_ can exceed 100 µg/m^3^ near streets with high traffic intensity. Expressing our results in terms of the 5th–95th percentile exposure contrast (25 μg/m^3^ NO_2_, 15,000 vehicles/day on the nearest street, or 7,200,000 vehicle-m/day within 100 m) thus reflects ranges that are frequently observed in European cities.

Our findings are consistent with previous reports of association of medium- to long-term exposure with subclinical atherosclerosis (Bauer et al. 2010; Diez Roux et al. 2008; Hoffmann et al. 2007; Künzli et al. 2005) and systemic inflammatory markers (Hoffmann et al. 2009a) although direct comparisons are limited given our use of NO_2_ instead of PM mass as a marker of pollution. However, our effect estimates are within the same order of magnitude as seen in previous studies.

In contrast, the association between pollution and ABI was less clear. Low ABI (< 0.9) is more clearly associated with mortality and cardiovascular disease than high ABI, but only high ABI was associated with traffic-related exposure in our study. Only 56 participants (2%) were classified as having low ABI [compared with 116 (4.2%) with high ABI], resulting in low power to detect an association. In the MESA study, 20-year exposures to PM_2.5_ and PM_10_ (PM ≤ 10 µm in diameter) were not associated with ABI as a continuous outcome, whereas 1-year mean PM_10_ exposures were associated with higher ABI, “indicating less subclinical disease among persons with greater exposure” (Diez Roux et al. 2008). On the other hand, a study in Germany reported that living 50 m from a major road compared to living > 200 m away was associated with an odds ratio (OR) of 1.77 (95% CI: 1.01, 2.1) for peripheral arterial disease (ABI of < 0.9 or history of treatment for peripheral artery disease), whereas no associations were found with annual residential PM_2.5_ (Hoffmann et al. 2009b).

The predictive power of an ABI of > 1.3 for cardiovascular risk has been less studied. It has been associated with calcification of the arterial wall, higher levels of many CVD risk factors (McDermott et al. 2005), and higher risk of all-cause mortality and foot ulcers; it has been weakly associated with heart failure and stroke (Allison et al. 2008). Thus, there is evidence of higher cardiovascular risk both at low and high levels of ABI. We found that traffic-related exposure was associated with high ABI. To our knowledge, the association of air pollution with high ABI has not been investigated previously. ABI is considered to be a marker for medial sclerosis, which is a specific form of arterial disease distinct from atherosclerosis (Alzamora et al. 2012). Because ABI is a ratio of systolic blood pressures, high ABI could result from low brachial pressure, or high ankle pressure, or both (Allison et al. 2008). Thus, if air pollution affects the upper and lower vascular beds differently, for example, by affecting brachial pressure at an earlier stage than ankle pressure, it could be associated with both high and low ABI. This hypothesis is consistent with our finding that high (vs. normal) ABI was not associated with IMT (adjusted RRR of 1.4; 95% CI: 0.3, 6.3 per 1 mm increase in IMTcca), whereas low (vs. normal) ABI was associated with thicker artery walls (adjusted RRR of 7.7; 95% CI: 1.6, 36.8, *p* = 0.01 per 1 mm increase in IMTcca).

In contrast to our findings, 20-year exposures to PM_2.5_ and PM_10_, in the MESA study were weakly associated with carotid IMT (1–3% differences in IMT for 12.5 and 21 µg/m^3^ increases in 20-year average PM_2.5_ and PM_10_, respectively) but not with ABI (Diez Roux 2008). In Los Angeles, a 10 µg/m^3^ increase in PM_2.5_ at the current address was associated with a 4.2% (95% CI: –0.2, 8.9) increase in IMT (Künzli et al. 2005). In a longitudinal study that included the population of Künzli et al. (2005), a 10 µg/m^3^ increase in PM_2.5_ and living within 100 m of a highway were associated with 2.5 µm/year (95% CI; –0.31, 5.38) and 5.5 µm/year (95% CI; 0.13, 10.8) increases in IMT, respectively (Künzli et al. 2010). In the Heinz Nixdorf Recall study, a 4.2 µg/m^3^ increase in 1-year average PM_2.5_ and a 1,939 m increase in distance from a major road were associated with a 4.3% (95% CI: 1.9, 6.7%) and a 1.2% (95% CI: –0.2, 2.6%) difference in IMT, respectively (Bauer et al. 2010). Distance to traffic was used as a marker of traffic-related exposure; thus a negative association with IMT was expected. A 5.2 µg/m^3^ increase in 1-year average PM_10_ was significantly associated with IMT in a study of > 2,000 civil servants in London (Tonne et al. 2012). Positive, though not significant, associations of IMT with measures of traffic intensity at the current address, but no association with a 25 µg/m^3^ increase in NO_2_, were observed in a population-based study of 745 young adults in Utrecht (Lenters et al. 2010).

Although associations with traffic as a surrogate of exposure appear to be more consistent across studies than associations with pollutants, more research is needed to clarify the role of specific constituents of air pollution on atherogenesis. In the study of young adults in Utrecht, associations of NO_2_, black smoke, PM_2.5_, and sulfur dioxide (SO_2_) with IMT, pulse wave velocity, and augmentation index were investigated, but significant associations were found only for NO_2_ with pulse wave velocity and augmentation index and for SO_2_ with augmentation index (Lenters et al. 2010). The evidence on the health effects of NO_2_ varies across studies. It has been reported to be more strongly associated with cardiovascular mortality than PM_2.5,_ black smoke, and SO_2_ (Beelen et al. 2008) and with cardiopulmonary mortality than PM_10_, total suspended particles, black smoke, and SO_2_ (Gehring et al. 2006), but Pope et al. (2002) found no association between NO_2_ and cardiopulmonary mortality. The different associations observed between NO_2_ and cardiopulmonary and cardiovascular outcomes across studies might reflect the suitability of using NO_2_ as a marker of traffic-related pollution. Although the general suitability of NO_2_ has been questioned before, the possibility of it being site dependent has not been studied. It has been reported that the correlation of NO_2_ with traffic intensity varies across locations (Raaschou-Nielsen et al. 2000). The correlation of NO_2_ with the components of the traffic emissions cocktail that promote (assuming the association was causal) or are truly associated with cardiovascular outcomes might also vary across locations.

Our exposure estimates (both NO_2_ and traffic) were based on data collected at participants’ residences, instead of data from air quality monitoring stations as far away as 10 km or more (Diez Roux et al. 2008). In addition, our traffic markers were based not only on proximity and road type classification (Allen et al. 2009; Hoffmann et al. 2009a), but also on actual traffic intensity derived from a dense traffic-count network. However, *R*^2^s for our NO_2_ models indicate some exposure measurement error (Basagaña et al. 2012). To evaluate the potential influence of measurement error (both bias and misclassification) introduced by the exposure models, we compared associations with our model-based estimates of 10-year average NO_2_ with associations based on annual mean NO_2_ at the closest monitor. Results (not shown) remained unchanged, suggesting that measurement error introduced by the model was negligible or comparable to the error associated with measures taken at the closest monitor.

Stronger positive associations in persons with a high education level were consistent across IMT measurements and across all markers of pollution. Larger estimated effects of traffic-related exposure on IMT were observed in men ≥ 60 years of age in all models. We did not find evidence of effect modification by Mediterranean diet, established risk factors for CVDs, or subclinical disease (indicated by medication treatment). Other studies have found heterogeneous associations across subgroups of age, sex, BMI, smoking status, socioeconomic status, town of residence, and other cardiovascular risks factors (Bauer et al. 2010; Künzli et al. 2005; Lenters et al. 2010). The detection of interactions in epidemiologic studies is often underpowered, and testing many interactions can lead to a multiple comparison problem. However, our subgroup analysis was designed a priori, and although our results may be subject to the aforementioned problems, the accumulated evidence over different studies will help to identify susceptible subgroups.

In Girona, exposure to traffic-related pollutants was higher for persons with a high education level. Higher NO_2_ concentrations were also found at the most privileged census-tract locations [see Supplemental Material, Figure S4 (http://dx.doi.org/10.1289/ehp.1205146)]. This has been reported before for southern European cities (Cesaroni et al. 2010), where wealthy persons live in downtown areas that are more polluted, in sharp contrast to what has been observed in North America (Gunier et al. 2003) and northern Europe (Chaix et al. 2006), where the most deprived bear the highest air pollution concentrations.

The persons with higher levels of education were also younger, had lower blood pressure, BMI, glucose levels, and low-density lipoprotein levels; a higher percent had quit smoking; and a lower percent had never smoked compared to persons with low and medium education levels. Stronger associations between air pollution and IMT among those with high versus low education levels were not explained by age interactions. This may indicate higher susceptibility but, more likely, a better detectability among persons with fewer competing risks for atherosclerosis (due to less confounding). It is difficult to evaluate whether this is supported by previous studies given that results are very heterogeneous. No effect modification by education level has been observed before for the association of PM_2.5_ with IMT, ABI, or coronary artery calcification (Diez Roux et al. 2008), nor for the association of NO_2_ or PM_2.5_ with IMT and arterial stiffness (Lenters et al. 2010). Whereas increasing effects of roadway proximity on aortic artery calcification have been reported for increasing income (*p*_trend_ < 0.01) (Allen et al. 2009).

Associations of PM_2.5_ with systemic inflammation markers have been reported to be stronger in men, and more specifically in highly educated men, in Germany (Hoffmann 2009a). Slightly stronger associations with annual PM_10_ were estimated among men compared to women in the study of civil servants in London (Tonne et al. 2012). Contrary to our results for men ≥ 60 years of age, stronger associations between PM_2.5_ and IMT were estimated for women ≥ 60 years of age in the study in Los Angeles (Künzli et al. 2005). The difference in augmentation index associated with increased levels of NO_2_ and PM_2.5_ was also higher for young women in Utrecht (Lenters et al. 2010).

Associations with NO_2_ or traffic load or intensity at the current address (at the time of examination) were weaker and less precise (data not shown) than associations with 10-year weighted averages across addresses, indicating exposure misclassification. Similar results were observed in the London study in which exposure at current address gave less precise estimates compared to exposure averaged during 1 year before examination (Tonne et al. 2012). This highlights the relevance of using long-term exposure when studying effects on the IMT. On the other hand, our results remained unchanged when using exposure at the address of longest residence compared with 10-year averaged exposure (data not shown). Thus, for settings similar to the Spanish Mediterranean region, in terms of patterns of spatial distribution of NO_2_ and low residential mobility (i.e., 80% of persons did not change address in the 10 years), the exposure at the address of longest residence may be a good proxy for long-term exposure.

Limitations of our study include the cross-sectional design and the possibility of unmeasured confounding, including confounding related to environmental tobacco smoke, and the lack of information on daily activity patterns to assess time spent at home. Strengths include the large population-based sample size and the availability of data on health and potential confounders, the 10-year address histories used to estimate long-term NO_2_ and traffic exposure markers, and the dense NO_2_ and traffic monitoring network data used to estimate exposures at each participant’s residences.

## Conclusions

We found evidence supporting an association between long-term exposure to traffic and subclinical carotid atherosclerosis and high ABI levels in our study population, with stronger associations in persons with a high level of education and in men ≥ 60 years of age. Longitudinal studies are needed to confirm whether air pollution contributes to the chronic processes of atherogenesis.

## Supplemental Material

(995 KB) PDF
