# Supplementary material for: Association between Long-Term Exposure to Traffic-Related Air Pollution and Subclinical Atherosclerosis: The REGICOR Study
Source: Environ Health Perspect. 2012 Dec 12;121(2):223–30. doi: 10.1289/ehp.1205146 (PMC3569680; doi:10.1289/ehp.1205146)

Supplemental Material

Association between Long-Term Exposure to Traffic-Related Air Pollution and Subclinical Atherosclerosis: The REGICOR Study

Authors:

Marcela Rivera, Xavier Basagaña, Inmaculada Aguilera, Maria Foraster, David Agis, Eric de Groot, Laura Perez, Michelle A. Mendez, Laura Bouso, Jaume Targa, Rafael Ramos, Joan Sala, Jaume Marrugat, Roberto Elosua, Nino Künzli

Table of Contents

Table S1. Variables and performance of land use regression models for each group of towns. 3

Table S2. Additional descriptive statistics of the study population included in main analyses....4

Table S3. Descriptive statistics of individually assigned exposure to air pollution and traffic. 5

Table S4. Correlations between markers of exposure to traffic-related pollutants 6

Table S5. Mean of individually assigned exposure to air pollution and traffic by group of towns. 7

Figure S1. Map of study region and locations of study participants. Points represent address of longest residence. 8

Figure S2. Scatter plot of concentrations derived with the models vs. concentrations at the measurement locations for participants living within 200m of a measurement location. 9

Figure S3. Subgroup analysis: Effect estimates of percent difference in intima media thickness (IMTcca and IMT6seg) associated with exposure contrasts between the 5th and 95th percentiles for 10-year weighted average values of NO_2 (25 ug/m3), traffic load within 100m (7,200,000 vehicle-meters/day), and traffic intensity on the nearest street (15,000 vehicles/day) according to adherence to Mediterranean diet. 10

Figure S4. Scatter plot of residential NO_2 (1-yr average across the participant addresses in each census tract) and deprivation index (factor analysis of the percentages of manual workers, unemployed and young (16-29 y.o.) people with low education in each census tract according to 2001 census. A higher the index indicates lower socioeconomic status) at the census tract level. Both measured at the address of longest residence...12

Table S1. Variables and performance of land use regression models for each group of towns.

Town(s) *(number of monitoring sites)*	Variables of land use regresion model	R^2_A	$R^2(cv)$
Girona Salt (N=151)	Building's floor number<br>24 hour total traffic load of all roads in 25m<br>24 hour total traffic load of all roads in 500m (excluding traffic load in 25m)<br>length of all roads within 1000m	0.63	0.61
Banyoles Porqueres (N=56)	24 hour total traffic load of all roads in 300m<br>product of traffic intensity on nearest road and inverse distance to nearest road<br>nitrogen oxide concentration derived from a regional dispersion model at 1x1 km resolution	0.33	0.32
Blanes (N=91)	24 hour total traffic load of major roads in 500m<br>length of all roads within 100m<br>24 hour total traffic intensity on nearest road<br>area of high density residential land within 100m	0.38	0.36
La Bisbal Palafrugell Palamós (N=105)	household density within 1000m<br>24 hour total traffic load of all roads in 50m<br>area of industrial land within 1000m	0.44	0.42
Olot Sant Joan (N=88)	length of all roads within 1000m<br>distance to major road<br>24 hour total traffic load of all roads in 25m<br>area of buildings within 100m<br>area of industrial land within 300m	0.53	0.50
Llagostera Sta.Cristina (N=43)	24 hour total traffic load of all roads in 500m	0.51	0.51

R^2_A: adjusted coefficient of determination

$R^2(cv)$: cross-validation coefficient of determination

Table S2. Additional descriptive statistics of the study population included in main analyses

n=2780. N (%) unless otherwise indicated

**Characteristic**	**N (%) unless otherwise indicated**
**Menopause (% in women)**	1075 (38.7)
**Hypertension†**	1265 (45.5)
**Diabetes mellitus**	349 (12.6)
**Hypercholesterolemia**	690 (24.8)
**Town of residence**	
Girona, Salt	1444 (51,9)
Blanes	290 (10,4)
Sant Joan les Fonts, Olot	185 (6,6)
Santa Cristina, Llagostera	272 (9,8)
Palamos, La Bisbal, Palafrugell	332 (11,9)
Porqueres, Banyoles	257 (9,2)

† Systolic blood pressure >=140 mm Hg or diastolic blood pressure >= 90 mm Hg. or treatment

Table S3. Descriptive statistics of individually assigned exposure to air pollution and traffic.

n=2780. Minimum, 5th percentile, mean, standard deviation, 95th percentile and maximum.

Exposure measurement	min	5th perc.	mean	sd	95th perc.	max
10yr. average NO_2 (µg/m^3)	4.9	9.7	20.7	8.4	35.5	47.7
NO_2 at address of longest residence (µg/m^3)	3.5	10.3	22.0	9.0	37.9	50.4
NO_2 at current address (µg/m^3)*	3.5	10.2	21.9	9.0	37.7	50.4
NO_2 at the closest monitor within 200m (µg/m^3)†	3.7	9.2	22.3	9.3	40.5	52.9
10yr. average traffic load 100m buffer (1000 veh m/day)	0	231	2551	2246	7436	11149
10yr. average traffic intensity nearest street (1000 veh/day)	0	0.1	3.2	5.4	15.2	34.2

* Sample size restricted to participants living in study area at moment of examination N=2723

† Sample size restricted to participant living within 200m of monitoring site N=2265. NO_2 was monitored with passive samplers

Table S4. Correlations between markers of exposure to traffic-related pollutants

n=2780. All correlation coefficients are significant at the 0.001 p-value level.

Exposure Measurement	10yr averaged NO2 (ug/m3)	NO2 at address of longest residence (ug/m3)	NO2 at current address (µg/m3)*	NO2 at the closest monitor within 200m (µg/m3)†	10yr. average traffic load 100m buffer (1000 veh m/day)	10yr. average traffic int. nearest st.(1000 veh/day)
10yr averaged NO2 (µg/m3)	1					
NO2 at address of longest residence (µg/m3)	0.990	1				
NO2 at current address (µg/m3)*	0.986	0.979	1			
NO2 at the closest monitor within 200m (µg/m3)†	0.770	0.780	0.761	1		
10yr. average traffic load 100m buffer (1000 veh m/day)	0.720	0.720	0.713	0.570	1	
10yr. average traffic int. nearest st.(1000 veh/day)	0.520	0.520	0.514	0.360	0.580	1

* Sample size restricted to participants living in study area at moment of examination N=2723

† Sample size restricted to participant living within 200m of monitoring site N=2265. NO_2 was monitored with passive samplers

Table S5. Mean of individually assigned exposure to air pollution and traffic by group of towns.

n=2780.

Town of residence	10yr averaged NO2 (ug/m3)	NO2 at address of longest residence (ug/m3)	NO2 at current address (µg/m3)*	10yr. average traffic load 100m buffer (1000 veh m/day)	10yr. average traffic int. nearest st.(1000 veh/day)
Girona, Salt	25.7	27.2	27.1	3572	4.7
Porqueres, Banyoles	14.1	15.0	15.0	1247	1.7
Blanes	23.9	25.6	25.6	2965	2.3
Palamos, La Bisbal, Palafrugell	12.9	13.7	13.7	1664	1.8
Sant Joan les Fonts, Olot	16.4	17.5	17.5	816	0.9
Santa Cristina, Llagostera	11.7	12.4	12.4	1164	1.7

Figure S1. Map of study region and locations of study participants. Points represent address of longest residence.

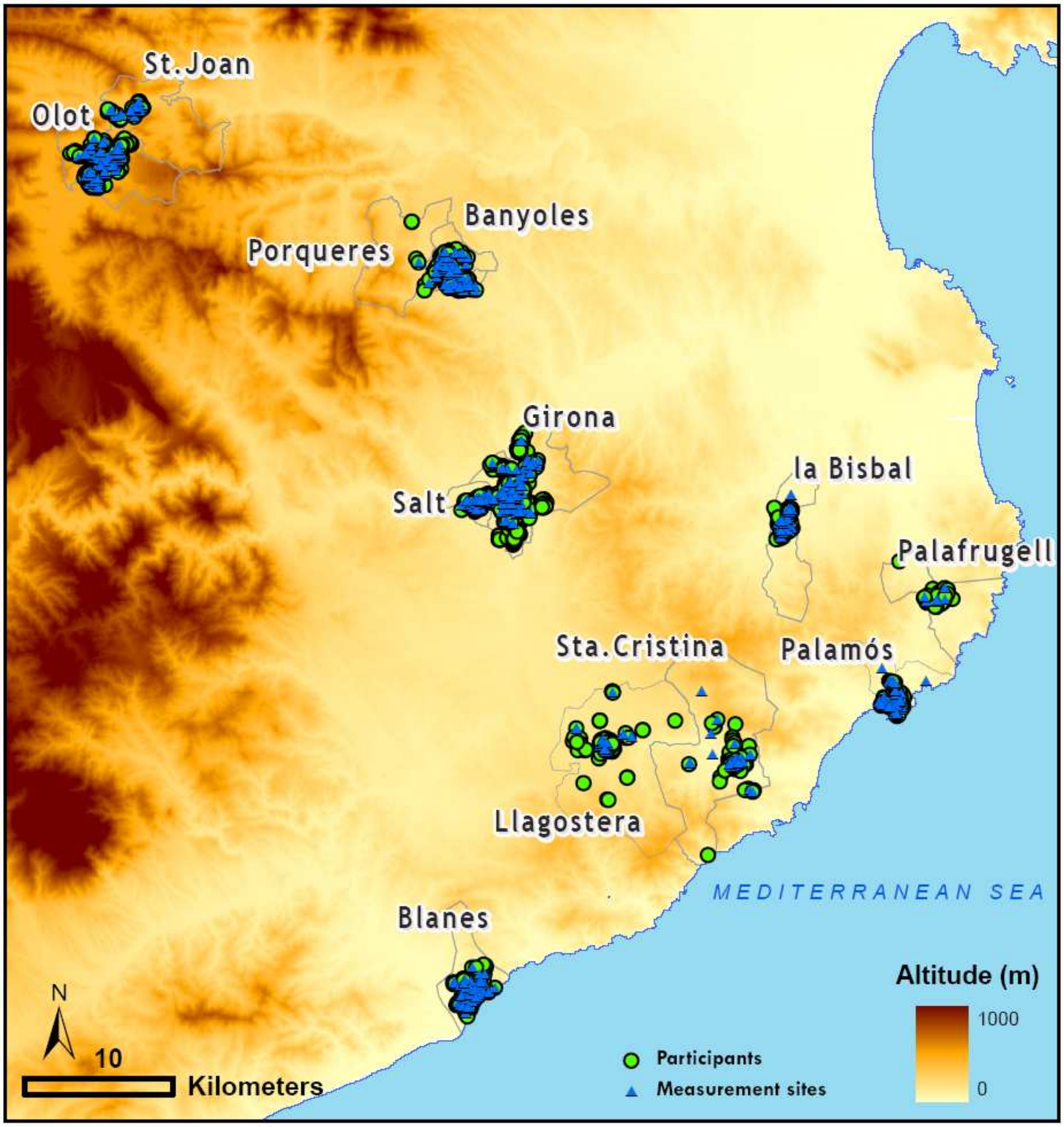

Figure S2. Scatter plot of concentrations derived with the models vs. concentrations at the measurement locations for participants living within 200m of a measurement location.

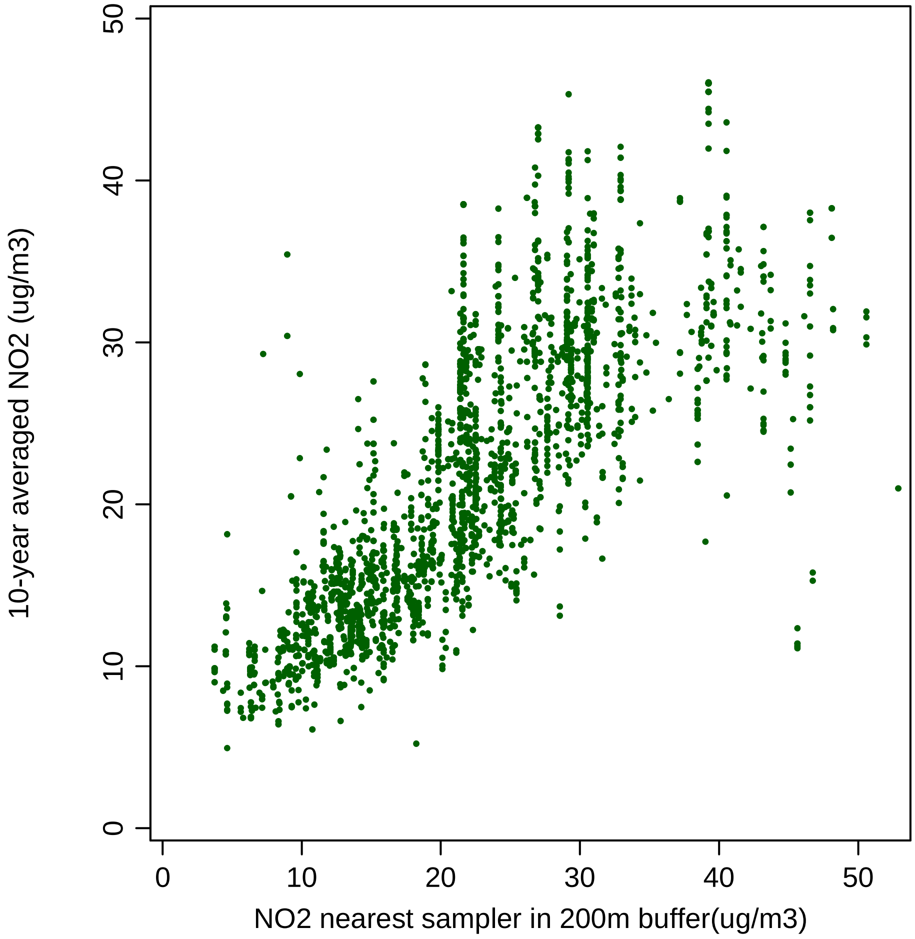

Figure S3. Subgroup analysis: Effect estimates of percent difference in intima media thickness (IMTcca and IMT6seg) associated with exposure contrasts between the 5th and 95th percentiles for 10-year weighted average values of NO_2 (25 ug/m3), traffic load within 100m (7,200,000 vehicle-meters/day), and traffic intensity on the nearest street (15,000 vehicles/day) according to adherence to Mediterranean diet.

Estimates adjusted by sex, age, sex-age interaction, smoking status, education, marital status, BMI, HDL, waist circumference, systolic and diastolic blood pressure, weekly energy expenditure in physical activity during leisure-time (tertiles), plausibility of reported diet, medication treatment and percentage of people with low education at the census tract level. Models for traffic load were additionally adjusted for occupational status.

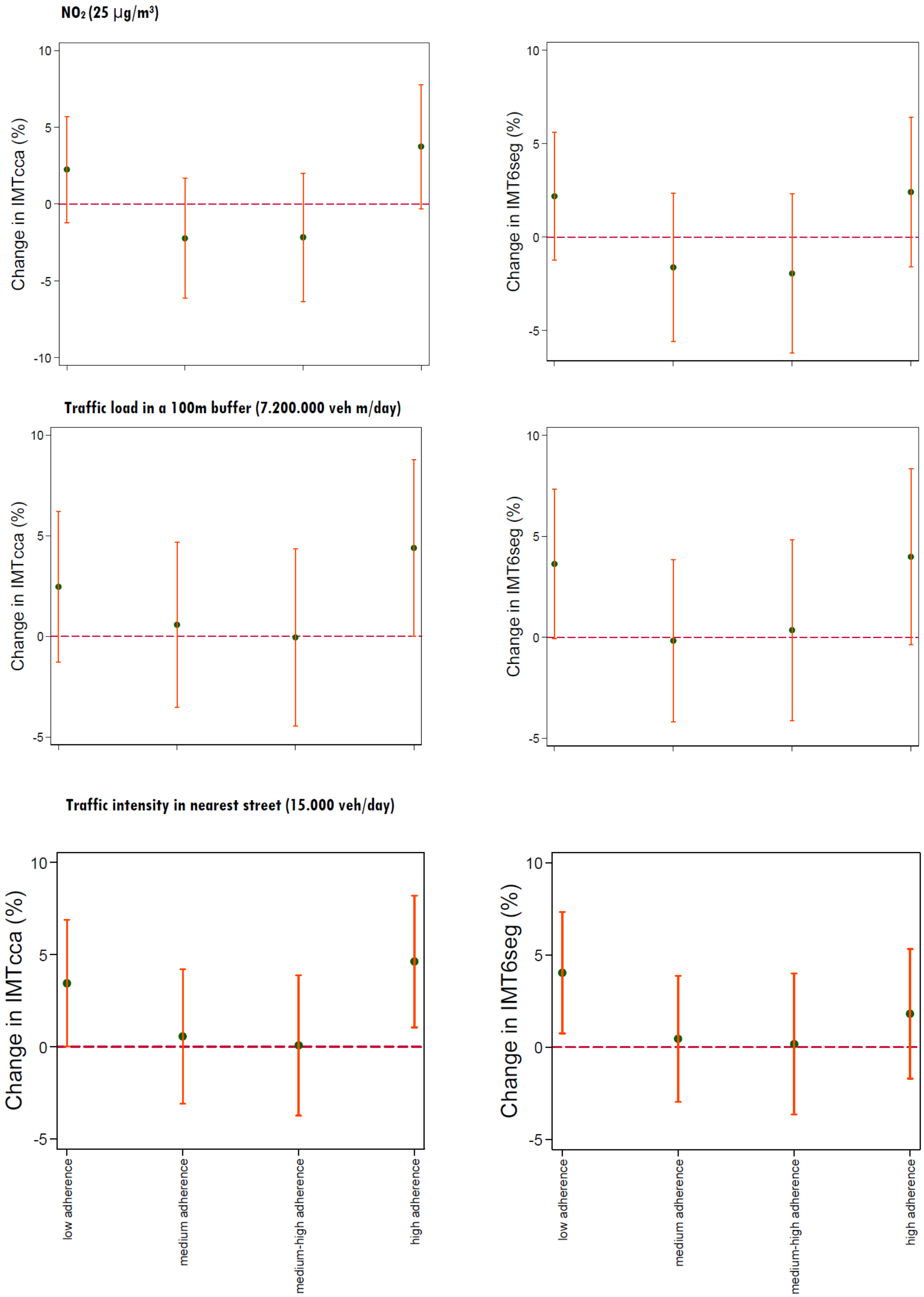
NO₂ (25 µg/m³)
Traffic load in a 100m buffer (7.200.000 veh m/day)
Traffic intensity in nearest street (15.000 veh/day)
Change in IMTcca (%)
Change in IMT6seg (%)
10
5
0
-5
-10
low adherence
medium adherence
medium-high adherence
high adherence

Figure S4. Scatter plot of residential NO_2 (1-yr average across the participant addresses in each census tract) and deprivation index (factor analysis of the percentages of manual workers, unemployed and young (16-29 y.o.) people with low education in each census tract according to 2001 census. A higher the index indicates lower socioeconomic status) at the census tract level. Both measured at the address of longest residence.

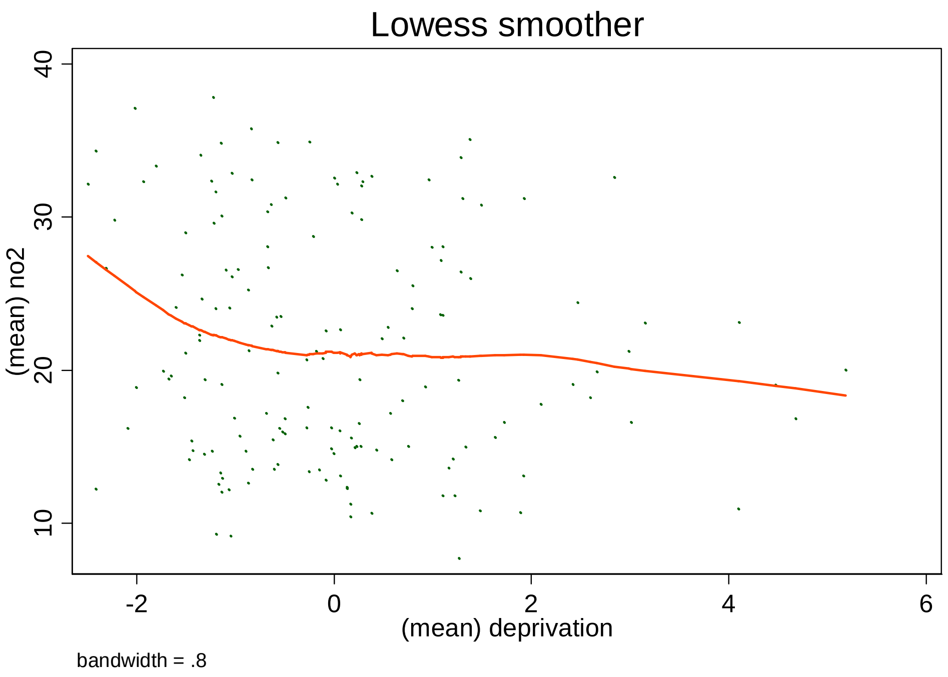

Supplement: (995 KB) PDF [file ehp.1205146.s001.pdf]
